# Sulfur disproportionation occurs globally across anoxic habitats and has multiple mechanisms of independent evolutionary origin

**DOI:** 10.1093/ismejo/wrag042

**Published:** 2026-03-02

**Authors:** Lukas V F Novak, Lijing Jiang, Marie Hemon, Marilina Fernandez, Léa Russo, Shasha Wang, Zongze Shao, Violette Da Cunha, Karine Alain

**Affiliations:** Univ Brest, CNRS, Ifremer, EMR 6002 BIOMEX, BEEP, IUEM, Plouzané F-29280, Finistère, France; Key Laboratory of Marine Genetic Resources, Third Institute of Oceanography, Ministry of Natural Resources of PR China, LIA/IRP 1211 MicrobSea, Sino-French International Laboratory of Deep-Sea Microbiology, Xiamen 361005, Fujian, PR China; Univ Brest, CNRS, Ifremer, EMR 6002 BIOMEX, BEEP, IUEM, Plouzané F-29280, Finistère, France; Université Paris-Saclay, INRAE, AgroParisTech, Micalis Institute, PAPPSO Platform, Jouy-en-Josas 78350, Yvelines, France; Univ Brest, CNRS, Ifremer, EMR 6002 BIOMEX, BEEP, IUEM, Plouzané F-29280, Finistère, France; Key Laboratory of Marine Genetic Resources, Third Institute of Oceanography, Ministry of Natural Resources of PR China, LIA/IRP 1211 MicrobSea, Sino-French International Laboratory of Deep-Sea Microbiology, Xiamen 361005, Fujian, PR China; Key Laboratory of Marine Genetic Resources, Third Institute of Oceanography, Ministry of Natural Resources of PR China, LIA/IRP 1211 MicrobSea, Sino-French International Laboratory of Deep-Sea Microbiology, Xiamen 361005, Fujian, PR China; Génomique Métabolique, Genoscope, Institut François Jacob, CEA, CNRS, Univ Evry, Université Paris-Saclay, Evry 91000, Essonne, France; Univ Brest, CNRS, Ifremer, EMR 6002 BIOMEX, BEEP, IUEM, Plouzané F-29280, Finistère, France

**Keywords:** microbial sulfur disproportionation, sulfur cycle, *Desulfobacterota*, genomics, phylogeny

## Abstract

Microbial sulfur disproportionation is a unique and enigmatic pathway of energy metabolism in bacteria where a single intermediate sulfur species, e.g. elemental sulfur, is simultaneously oxidized and reduced while generating ATP. We do not have a complete picture of the molecular mechanisms underlying microbial sulfur disproportionation and several pathways are likely involved depending on the taxon. This impairs our ability to investigate the evolutionary history, antiquity, taxonomic distribution, and ecological significance of this metabolism. Here we provide a comprehensive overview of all previously proposed candidate genes, translation of some of which is upregulated under sulfur disproportionation conditions, as well as other sulfur-utilizing dissimilatory metabolic pathways, across the diversity of all genomically characterized sulfur-disproportionating bacteria from a wide range of environments, and phylogenetically reconstruct their evolutionary history. We conclude that the MOLY cluster of likely extracellular molybdopterin oxidoreductases and the YTD cluster of mostly uncharacterized proteins are currently the best candidates for sulfur disproportionation markers in *Desulfobacterota* and *Nitrospirota*, and confirm previous observations that other taxa likely use different mechanisms. We also show that sulfur disproportionation pathways utilize enzymes from other processes of sulfur metabolism. The most parsimonious scenario for evolutionary origins of MOLY and YTD clusters is their presence already in the last common ancestor of *Desulfobacterota*, *Nitrospirota*, and *Acidobacteriota*, which lived in the Paleoarchean. Our analyses substantially narrow down the field of viable candidate genes and provide directions for future research.

## Introduction

Sulfur is present in a broad range of electron donors and acceptors in microbial energy metabolism. The various oxidative, reductive, and disproportionation reactions performed by microbes, together with the assimilation of sulfur into organic compounds and abiotic processes, form the biogeochemical sulfur cycle [1–4]. Almost all the energy-conserving dissimilatory pathways of the sulfur cycle are of the respiratory type, i.e. they require two different types of molecules, one undergoing oxidation and the other reduction. Sulfur can play both roles due to its wide spectrum of oxidation states. However, an additional electron donor or acceptor is still required. This can be organic matter for sulfate reducers or molecular oxygen (O_2_) for sulfide oxidizers. In contrast, microbial sulfur disproportionation (MSD) is analogous to organic fermentation in that it requires only a single type of input molecule that acts both as electron donor and acceptor within a single process [5]. This molecule can be one of the sulfur species of intermediate oxidation state, like elemental sulfur (S^0^), sulfite (SO_3_^2−^), thiosulfate (S_2_O_3_^2−^), or rarely tetrathionate (S_4_O_6_^2−^). The oxidative branch of MSD leads to the production of sulfate (SO_4_^2−^) and possibly also sulfite [4], whereas the reductive branch leads to sulfide (H_2_S). Electrons are transported between these half-reactions and ATP is likely produced in whole or in part by substrate-level phosphorylation [6, 7]. MSD is distinct from the better understood O_2_-dependent sulfur disproportionation catalyzed by sulfur oxygenase reductase (Sor) in sulfur-oxidizing archaea [8], which is not discussed here.

The disproportionation of sulfite and thiosulfate has been known to enable microbial growth since its discovery in the 1980s [5, 7], but growth by MSD using elemental sulfur, which is endergonic under standard conditions [9], has mostly been documented in the last 2 decades. Recent thermodynamic investigations show that the three best-known substrates (sulfite, thiosulfate, and elemental sulfur) may be able to support microbial energy production (∆*G_r_* < 0) under natural conditions, e.g. in deep-sea hydrothermal vents or sulfidic karst caves [10, 11]. Multiple experimental studies have shown the ability of various bacterial strains to grow under solely S^0^-disproportionating conditions in vitro [12–16].

The ecological role of MSD has been most often studied in mesophilic oxygen-depleted marine and freshwater sediments, where it is responsible for a large part of sulfur cycling, metabolizing more than half of all thiosulfate present in studied estuarine sediments [17]. Sulfur-disproportionating bacteria have also been isolated from marine hydrothermal vents, where mixing of hydrothermal fluids with oxygenated seawater provides necessary intermediate sulfur species and high concentrations of metal oxides help scavenge sulfide, one of the end products of MSD, increasing the energy yield [6, 10]. Bacteria are able to grow using MSD also in environments devoid of sulfide scavenging minerals, as demonstrated by their thriving in soda lakes, where the high pH allows sulfide to be consumed in reactions with soluble polysulfides (S*_n_*^2−^) [14]. MSD is an important sink for microbially produced elemental sulfur in karst caves and may be a substantial player in speleogenesis, because it is the only known biotic process capable of lowering pH below the water table, contributing to weathering of carbonate rocks [11].

Anaerobic MSD capability has been experimentally demonstrated in bacterial strains from a variety of natural and anthropogenic environments, growing under a range of physico-chemical conditions, from moderate to elevated temperatures, at pH levels ranging from acidic to alkaline, and under a wide variety of chemical conditions [6]. Most of these strains are obligate anaerobes but there are also a few facultative aerobes capable of MSD, such as *Pantoea agglomerans* SP1 (*Pseudomonadota*), *Sulfurimonas marina*, and *Sulfurimonas hydrogeniphila* (*Campylobacterota*) [16]. Determining the full range of environmental conditions where MSD takes place is currently not possible due to the lack of reliably demonstrated molecular markers of MSD, i.e. gene(s) exclusively involved in this metabolism whose presence in a genome or environmental sequences could be used as a proxy for the presence of MSD itself. No molecular marker unique to MSD has been conclusively identified so far.

All known MSD-positive strains belong to the phyla *Desulfobacterota*, *Nitrospirota*, *Pseudomonadota*, *Campylobacterota*, and *Bacillota* [6, 18, 19], covering both *Gracilicutes* (proposed kingdom *Pseudomonadati*) and *Terrabacteria* (*Bacillati*), the two major divisions of the bacterial tree of life. Anaerobic MSD capability has not been observed in archaea or eukaryotes. It is not known whether MSD originated only once and then spread—either vertically or by lateral gene transfer (LGT)—to form the current diversity, or whether it had multiple independent origins. The presence of MSD in *Bacillota*, very distantly related to the other four phyla, suggests LGT or at least two independent origins. However, it is presently not possible to reject MSD capabilities in a broader diversity of microbes that would bridge this gap. The antiquity of MSD also remains unclear [20]. Different paleoenvironmental reconstructions based on the geological record of sulfur isotopes point towards a start of a significant presence of MSD either as early as 3.5 Ga in the Paleoarchean [21, 22] or as late as 1.3 Ga in the Mesoproterozoic [23].

Contemporary genomic and phylogenetic techniques could make it possible to answer questions concerning the ecology and evolution of MSD, in the same way as for the better characterized pathways of sulfate reduction or sulfur oxidation [24–26], if the molecular mechanisms and associated genes were better known. Unfortunately, although our knowledge of MSD pathways is not without clues, it is far from complete. The greatest hindrance to progress in this direction lies in the absence of reliable molecular marker(s), if any exist.

Previous works have demonstrated that there are necessarily multiple distinct pathways for MSD, depending on the used sulfur species and genomic context [6, 9, 13, 27]. Some of these pathways use enzymes from other sulfur cycle catabolic processes, such as Adenylylsulfate (APS) reductase AprAB, Sulfate adenylyltransferase Sat, Dissimilatory (bi)sulfite reductase subunits and co-substrate DsrAB/DsrC, Quinone-interacting membrane-bound oxidoreductase complex QmoABC, Thiosulfate reductase PhsABC, and Tetrathionate reductase TtrABC involved in reduction or oxidation of various sulfur compound in diverse prokaryotes [6, 28, 29]. The SorAB involved in sulfur oxidation could also be involved in MSD in *Campylobacterota* [27]. Proteomic analyses carried out with various strains of *Desulfobacterota* revealed that some of these enzymes were overexpressed under sulfur compound disproportionation conditions: AprAB, Sat, DsrAB, QmoABC, PhsABC, and TtrABC, [28, 29]. Other enzymes proposed to be involved in MSD, but without clearly established involvement in other catabolic pathways, include a small transmembrane protein DsrE2, a heterodimeric sulfide dehydrogenase SudhAB, and rhodanese-like sulfurtransferase SseA [6, 30, 31].

Multiple genes and gene clusters were proposed to be MSD-specific and therefore potentially serve as molecular markers of this metabolic process. A C-terminal truncated form of the adenylylsulfate reductase AprB (referred further as AprBt), an enzyme otherwise involved in sulfate reduction, was proposed based on its presence in sulfur compound disproportionating members of *Desulfobulbaceae* and *Dissulfurirhabdaceae* (*Desulfobacterota*) [32, 33]. A short EscU/YscU/HrcU family protein (referred further as Eyh [15]) was proposed based on comparative genomics of MSD-positive and negative strains of *Desulfobacterota* [34]. The YTD cluster was proposed based on comparative genomics of *Nitrospirota*, *Desulfobacterota*, and *Bacillota*. This cluster typically consists of a *yedE*-related gene, a *tusA* sulfurtransferase, a *dsrE*-related gene, and 2–3 conserved hypothetical proteins (referred further as *yedE*, *dsrE*, *tusA*, and *chp*1–3) [18]. This YTD cluster was shown to be overexpressed in *Desulfolithobacter dissulfuricans* (*Desulfobacterota*) during thiosulfate disproportionation [6, 28, 29], YedE was shown to be overexpressed in *Desulfurivibrio dismutans* (*Desulfobacterota*) during S^0^ disproportionation [29] and *tusA* was overexpressed in *Dissulfuribacter thermophilus* S69^T^ (*Desulfobacterota*) during thiosulfate disproportionation (*M. Fernandez*, personal communication). The MOLY cluster encoding molybdopterin reductase A and B subunits and a TorD/DmsD chaperone (referred further as MolyA, MolyB, and MolyC) was proposed based on comparative genomics of MSD-positive and negative strains of *Desulfobacterota* [15].

MSD often co-occurs with dissimilatory sulfate reduction, sulfur reduction, or sulfur oxidation in the studied bacteria [6, 19] and it was shown that sulfite and thiosulfate disproportionation is catalyzed by the sulfate reduction pathway operating in reverse direction in *Desulfobacterota* [7, 15, 28]. The previously proposed MSD mechanisms, using enzymes also involved in other metabolic pathways, can fully or almost fully explain the disproportionation of sulfite and thiosulfate. However, the additional step(s) needed for elemental sulfur disproportionation, arguably the most unique aspect of MSD, remain unknown, and so represent the best direction for the search for potential molecular markers of MSD. For this reason, we treat S^0^ disproportionators as a special functional category in this work, i.e. category "++" as described below. All genes previously proposed to be involved in MSD, and lacking clearly established roles in respiratory pathways, are referred here as "MSD candidates." All genes previously proposed to be exclusively involved in MSD are referred here as “MSD molecular marker candidates”.

In order to complement the ongoing experimental efforts into identifying the underlying mechanisms of MSD, we approached this question from an evolutionary point of view, employing computational approaches and utilizing recent advances in microbial genomics. We present a large-scale comparative genomic analysis, combined with an extensive literature review of experimentally demonstrated MSD capabilities across the diversity of bacteria. We compiled a database of all available genomes of the MSD-positive bacterial strains, as well as related strains demonstrated to be MSD-negative, and searched it for proposed MSD-related genes, both shared with other sulfur cycle dissimilatory pathways, as well as those potentially specific to MSD. We reconstructed phylogenetic trees for the candidate genes in order to reveal their evolutionary history across the bacterial tree of life and to investigate whether they can be used as potential molecular markers of MSD. This allowed us to select certain candidate genes as more likely to be involved in MSD, while rejecting others, and to propose an evolutionary scenario about the emergence of the most promising candidate genes and gene clusters.

## Materials and methods

### Proteomic datasets

Pure strain-sequenced genomic assemblies and predicted proteomes of the strains of interest, including the strains with positive experimental MSD evidence, strains with negative experimental MSD evidence, and strains without available experimental evidence of MSD (Table 1, Supplementary table 1) were downloaded from the NCBI GenBank (ncbi.nlm.nih.gov/genbank) or from JGI IMG/M (img.jgi.doe.gov) databases. The collection of datasets concluded on 15 April 2025. Only one newly published genome assembly was added after this date and not included in the phylogenetic analyses: the metagenome-assembled genome of *Exiguobacterium* sp. [35]. All genomic and proteomic data used in this work are based on previously published literature, as detailed in Table 1.

**Table 1:** List of bacterial strains with positive experimental evidence for MSD.

Phylum	Order	Family	Strain	MSD: S^0^	MSD: sulfite	MSD: thiosulfate	Reference for experimental evidence of MSD	Genome assembly
*Desulfobacterota*	*Thermodesulfobacteriales*	*Thermodesulfobacteriaceae*	*Caldimicrobium rimae* DS(T)	yes		no	Miroshnichenko et al., 2009	N/A
			*Caldimicrobium thiodismutans* TF1(T)	yes	yes	yes	Kojima et al., 2016	GCF_001548275.1
			*Thermosulfurimonas dismutans* S95(T)	yes	yes	yes	Slobodkin et al., 2012	GCF_001652585.1
			*Thermosulfurimonas marina* SU872(T)	yes	yes	yes	Frolova et al., 2018	GCF_001652585.1
			*Thermosulfurimonas* sp. F29	yes	no	yes	Allioux et al., 2022	GCF_019688735.1
			*Thermosulfuriphilus ammonigenes* ST65(T)	yes	yes	yes	Slobodkina et al., 2017	GCF_011207455.1
		*Thermodesulfatatoraceae*	*Thermodesulfatator atlanticus* AT1325(T)	yes	no	no	Allioux et al., 2021	GCF_000421585.1
	*Desulfobulbales*	*Desulfobulbaceae*	*Desulfobulbus elongatus* FP(T)	yes	no		Ward et al., 2021	GCF_000621145.1
			*Desulfobulbus oligotrophicus* Prop6(T)	no	yes	no	El Houari et al., 2017	GCF_016446285.1
			*Desulfobulbus propionicus* 1pr3(T)	yes	no	no	Lovley & Phillips, 1994	GCF_000186885.1
			*Desulfolithobacter dissulfuricans* GF1(T)	yes	no	yes	Hashimoto et al., 2022	GCF_025998535.1
			*Desulfurivibrio alkaliphilus* AHT 2(T)	yes			Poser et al., 2013	GCF_000092205.1
			*Desulfolithobacter dismutans* B35	yes			N/A	GCA_046599375.1
		*Desulfocapsaceae*	*Desulfocapsa* sp. Cad626	yes	yes	yes	Peduzzi et al., 2003	N/A
			*Desulfocapsa sulfexigens* SB164P1(T)	yes	yes	yes	Frederiksen & Finster, 2004	GCF_000341395.1
			*Desulfocapsa thiozymogenes* Bra2(T)	yes	yes	yes	Janssen et al., 1996	IMG: 2514885009
			*Desulfofustis glycolicus* PerGlyS(T)	yes	no	no	Finster, 2008	GCF_900130015.1
			*Desulforhopalus singaporensis* S'pore T1(T)		yes		Lie et al., 1999	GCF_900104445.1
			*Desulfobacterota* strain M19	yes			Hemon et al., 2025	GCA_048820955.1
		*Thiovibrionaceae*	*Thiovibrio frasassiensis* RS19–109(T)	yes	no	yes	Aronson et al., 2023	GCF_029607905.1
	*Desulfovibrionales*	*Desulfovibrionaceae*	*Desulfobaculum senezii* CVL(T)		yes	yes	Hsien Tsu et al., 1998	IMG: 2574179788
			*Desulfolutivibrio sulfodismutans* ThAcO1(T)	no	yes	yes	Bak & Pfennig, 1987	N/A
			*Desulfovibrio aminophilus* ALA-3(T)		yes*	yes*	Baena et al., 1998	GCF_000422565.1
			*Desulfovibrio cuneatus* STL 1(T)		yes	no	Sass et al., 1998	N/A
			*Desulfovibrio desulfuricans* CSN		yes	no	Cypionka et al., 1998	N/A
			*Desulfovibrio litoralis* STL 4		yes	no	Sass et al., 1998	N/A
			*Desulfovibrio oxyclinae* P1B(T)		yes	yes	Krekeler, et al., 1997	GCF_000375485.1
			*Desulfurivibrio* sp. AMeS2	yes			Poser et al., 2013	N/A
			*Humidesulfovibrio mexicanus* Lup 1(T)		yes	yes	Hernandez-Eugenio et al., 2000	GCF_900188225.1
			*Paucidesulfovibrio longus* DSM 6739(T)		yes	no	Magot et al., 1992	GCF_000420485.1
			*Salidesulfovibrio brasiliensis* LVform1(T)			yes	Warthmann et al., 2005	GCF_001311825.1
		*Desulfonatronaceae*	*Desulfonatronum lacustre* Z-7951(T)	no	yes	yes	Pikuta et al., 1998	GCF_000519265.1
			*Desulfonatronum parangueonense* PAR180(T)	no	yes	yes	Pérez Bernal et al., 2017	N/A
			*Desulfonatronum thioautotrophicum* ASO4–1(T)	no	yes	yes	Sorokin et al., 2011	GCF_000934745.1
			*Desulfonatronum thiodismutans* MLF 1(T)	no	yes	yes	Pikuta et al., 2003	GCF_000717475.1
			*Desulfonatronum thiosulfatophilum* ASO4–2(T)	no	yes	yes	Sorokin et al., 2011	GCF_900104215.1
		*Desulfonatronovibrionaceae*	*Desulfonatronovibrio hydrogenovorans* Z-7935(T)	yes	yes	yes	Sydow et al., 2002	GCF_963865035.1
			*Desulfonatronospira delicata* AHT 6(T)	no	yes	yes	Sorokin et al., 2008	N/A
			*Desulfonatronospira sulfatiphila* ASO3–2(T)		yes	no	Sorokin & Chernyh, 2017	N/A
			*Desulfonatronospira thiodismutans* AHT 8	no	yes	yes	Sorokin et al., 2008	N/A
			*Desulfonatronospira thiodismutans* ASO3–1(T)	no	yes	yes	Sorokin et al., 2008	GCF_000174435.1
			*Desulfonatronovibrio magnus* AHT 22(T)	no	yes	yes	Sorokin et al., 2011	GCF_000934755.1
	*Desulfobacterales*	*Desulfobacteraceae*	*Desulfobacter curvatus* AcRM3(T)		no	yes*	Krämer & Cypionka, 1989	GCF_000373985.1
			*Desulfobacter hydrogenophilus* AcRS1(T)		no	yes*	Krämer & Cypionka, 1989	GCF_004319545.1
		*Desulfococcaceae*	*Desulfococcus multivorans* Göttingen (1be1)(T)		no	yes*	Krämer & Cypionka, 1989	GCF_002009335.2
	*Desulfomonilales*	*Desulfomonilaceae*	*Desulfomonile tiedjei* DCB-1(T)			yes	Mohn & Tiedje, 1990	GCF_000266945.1
	*Desulfatiglandales*	*Desulfatiglandaceae*	*Desulfatiglans anilini* Ani 1(T)			yes*	Schnell et al., 1989	GCF_000422285.1
	*Dissulfuribacterales*	*Dissulfuribacteraceae*	*Dissulfuribacter thermophilus* S69(T)	yes	yes	yes	Slobodkin et al., 2013	GCF_001687335.1
	*incertae sedis*	*Dissulfurirhabdaceae*	*Dissulfurirhabdus thermomarina* SH388(T)	yes	yes	no	Slobodkina et al., 2016	GCF_012979235.1
		*incertae sedis*	*Dissulfurimicrobium hydrothermale* Sh68(T)	yes	yes	yes	Slobodkin et al., 2016	GCF_022026155.1
*Nitrospirota*	*Thermodesulfovibrionales*	*Dissulfurispiraceae*	*Dissulfurispira thermophila* T55J(T)	yes	no	yes	Umezawa et al., 2021	GCF_014701235.1
*Campylobacterota*	*Campylobacterales*	*Sulfurimonadaceae*	*Sulfurimonas hydrogeniphila* NW10(T)	no	no	yes	Wang et al., 2022	GCF_009068765.1
			*Sulfurimonas marina* B 2(T)	no		yes	Wang et al., 2022	GCF_014905095.1
			*Sulfurimonas* sp. HSL1–2	yes		yes	Wang et al., 2022	GCF_039645565.1
			*Sulfurimonas* sp. HSL1–6	yes		yes	Wang et al., 2022	GCF_039645955.1
			*Sulfurimonas* sp. HSL3–1	yes		yes	Wang et al., 2022	GCF_039645995.1
			*Sulfurimonas* sp. HSL3–2	yes		yes	Wang et al., 2022	GCF_039645965.1
			*Sulfurimonas* sp. HSL3–7	yes		yes	Wang et al., 2022	GCF_039645985.1
			*Sulfurimonas* sp. ST-25	yes		yes	Wang et al., 2022	GCF_048079205.1
			*Sulfurimonas* sp. ST-27	yes		yes	Wang et al., 2022	GCA_048081595.1
			*Sulfurimonas* sp. NWX367	yes		yes	Wang et al., 2022	GCF_039667065.1
			*Sulfurimonas* sp. NWX79	yes		yes	Wang et al., 2022	GCF_039667035.1
		*Sulfurovaceae*	*Sulfurovum riftiae* 1812E(T)	yes	no	yes	Wang et al., 2022	GCF_001595645.1
			*Sulfurovum* sp. HSL1–3 ST1–3	yes		yes	Wang et al., 2022	GCF_020911965.1
			*Sulfurovum* sp. ST-21	yes		yes	Wang et al., 2022	GCF_048079285.1
			*Sulfurovum* sp. ST-29	yes		yes	Wang et al., 2022	PRJNA1222866
	*Desulfurellales*	*Desulfurellaceae*	*Desulfurella amilsii* TR1(T)	yes			Florentino et al., 2016	GCF_002119425.1
*Bacillota*	*Dethiobacterales*	*Dethiobacteraceae*	*Dethiobacter alkaliphilus* AHT 1(T)	yes			Poser et al., 2013	GCF_000174415.1
	*Eubacteriales*	*Desulfotomaculaceae*	*Desulfotomaculum nigrificans* DSM 574(T)	no	no	yes*	Krämer & Cypionka, 1989	GCF_000189755.2
			*Desulfofundulus thermobenzoicus* TSB(T)			yes	Jackson & McInerney, 2000	N/A
		*Peptococcaceae*	*Desulfofundulus salinus* 435			yes	Nazina et al., 2005	GCF_003627965.1
			*Desulfotomaculum* sp. 781			yes	Nazina et al., 2024	N/A
	*Bacillales*	*incertae sedis*	*Exiguobacterium* sp.	yes			Wu et al., 2025	SRR30630613
*Pseudomonadota*	*Enterobacterales*	*Erwiniaceae*	*Pantoea agglomerans* SP1	yes			Obraztsova et al., 2002	N/A

^*^Disproportionation without growth

(T) stands for type strain.

The completeness and redundancy of the assemblies were assessed using the CheckM2 1.1.0 tool [36] implemented in Galaxy Europe 25.0.5 [37]. Based on the results (Supplementary table 1), one strain of interest without any evidence for or against MSD was eliminated from further analyses. When a predicted proteome was not available, an ad hoc proteome was predicted using Prodigal 2.6.3 [38]. All 111 proteomes were kept as separate multi-FASTA files for later sequence similarity searches using profile hidden Markov models (profile HMMs) [39].

### HMM profiles

Where possible, the relevant publicly available HMM profiles were downloaded from the InterPro database [40]. For certain genes involved in sulfate and sulfite reduction, without publicly available HMM profiles, the multiple amino acid sequence datasets published in previous work [25] were used to build new HMM profiles, as described below.

For the rest of the genes of interest, including the generally poorly characterized candidates for MSD molecular markers, new HMM profiles were constructed completely de novo. Specific amino acid sequences listed in the original publications, discussing these genes in the context of MSD, were downloaded from the NCBI GenBank database and used as a seed of the dataset for building new HMM profiles. These were further complemented by a larger number of sequences from the four bacterial phyla of interest (*Desulfobacterota*, *Campylobacterota*, *Bacillota*, and *Nitrospirota*) selected based on high sequence similarity to seed sequences. All the used sequences, together with the relevant references and type of evidence used for including them, are listed in Supplementary table 2. All HMM profiles created for this work are deposited in Supplementary file 1.

### HMM search

The amino acid sequences were aligned using MAFFT 7.520 [41] with default settings and as untrimmed alignments used for generating HMM profiles by the hmmbuild tool of HMMER 3.4 [39]. These profiles were searched against the 111 predicted proteomes by the hmmsearch tool of HMMER 3.4. Results of the searches (Supplementary file 1) were manually inspected and an *E*-value cutoff value (Supplementary table 1) was determined based on original annotations and results of NCBI BLASTp against the nr database [42]. The distribution of sequence lengths, as well as domain architectures estimated using the online NCBI BLASTp tool, were used in determining the *E*-value cutoff value. In the cases of YedE, TusA, MolyA, MolyB, MolyC, PhsA, and TtrA, the *E*-value cutoff values were further corroborated based on results of phylogenetic analyses, as discussed below. The results that passed the cutoffs are listed in Supplementary table 1 and their amino acid sequences were gathered in single-gene multi-FASTA files. Where appropriate, the results were validated using DiSCo [43] (not shown).

### Phylogeny

In order to detect potential evidence of lateral gene transfer (LGT) in the phylogenetic trees, further homologous sequences were gathered by a BLASTp search with each sequence from every single-gene FASTA file as a query against the NCBI nr database with an *E*-value cutoff of 10^−10^ and maximum 10 target sequences. Furthermore, additional sequences were gathered by taxon-restricted BLASTp searches against the NCBI nr database targeting individual prokaryotic phyla (Supplementary table 3) in order to represent a broad taxonomic diversity of sequences of the genes of interest. All resulting sequences for a single gene were aligned using MAFFT 7.520 [41] and automatically trimmed using trimAl 1.2rev59 (−gt 0.7) [44]. Maximum likelihood phylogenetic trees were reconstructed using IQ-TREE 2.2.2.6 with LG + C60 + G + F substitution model and 1000× ultrafast bootstraps [45]. Alternative statistical support values were calculated using the SH-like approximate likelihood ratio test implemented in IQ-TREE 2.2.2.6 [46, 47]. Excessively divergent sequences forming long branches were removed from the original datasets and the aligning, trimming and tree reconstruction steps were repeated. Trees were visualized and edited using TreeViewer 2.1.0 [48]. Statistical support values from the two methods were combined using a custom Python script and manually included in the final tree figures (Supplementary file 3) using Inkscape 1.3. For SH-like approximate likelihood ratio test, only values of at least 50% were included. In trees with large number of putative orthologous groups, the SH-like approximate likelihood ratio test values were include only in OC1. Complete statistical support information can be found in Supplementary file 4.

### Miscellaneous

Subcellular localization of proteins was estimated using DeepTMHMM 1.0 [49]. The genomic context of the genes of interest was investigated by visualizing them using IGV 2.9.4 [50] and the conserved syntenic blocks were plotted manually using Inkscape 1.3 (Supplementary file 2). All alignments and raw phylogenetic trees are given in Supplementary file 4.

## Results and discussion

### Microbial sulfur disproportionation across bacterial diversity

**Figure 1:**
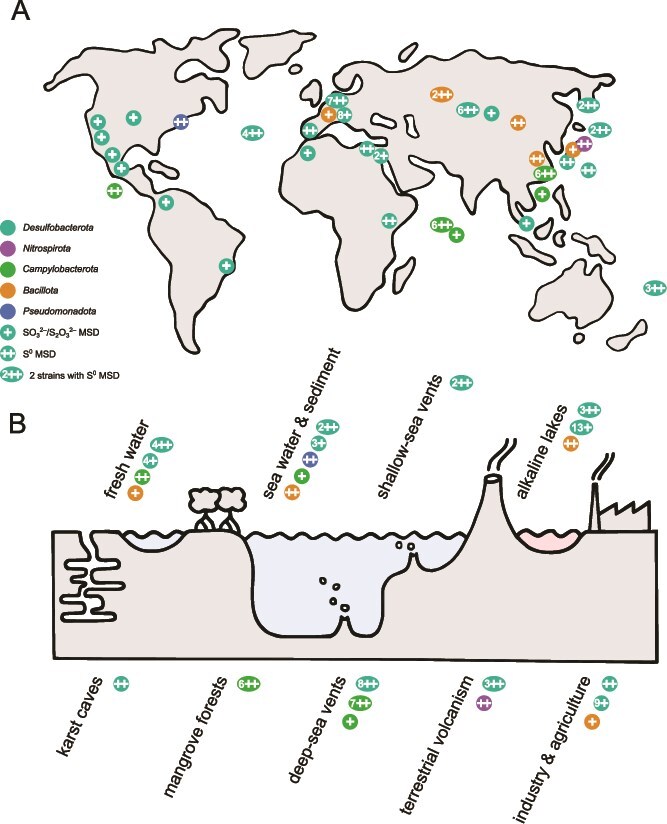
Distribution of MSD-positive strains across geography and habitats. (A) Global map of sampling sites for the MSD-positive strains. Cyan: *Desulfobacterota*, magenta: *Nitrospirota*, green: *Campylobacterota*, Orange: *Bacillota*, blue: *Pseudomonadota*. Numbers indicate multiple strains isolated from similar geographic locations. Double plus indicates the category "++," i.e. strains capable of elemental sulfur disproportionation, both with and without the ability to disproportionate sulfite and/or thiosulfate. Single plus indicates the category "+," i.e. strains capable of sulfite and/or thiosulfite disproportionation. (B) Habitats of the MSD-positive strains.

After literature search, we identified a list of 74 strains with positive experimental evidence of MSD [7, 9, 12–16, 19, 28, 35, 48, 51–89]. All belong to one of the five bacterial phyla known to include taxa capable of MSD: 50 strains belong to the *Desulfobacterota*, 16 to *Campylobacterota*, six to *Bacillota*, one to *Nitrospirota*, and one to *Pseudomonadota*. Each phylum contains strains capable of disproportionating S^0^ (Table 1). The strains have been isolated from a broad spectrum of natural and artificial habitats across five continents and three oceans (Fig. 1A), with the largest number of 22 coming from various hydrothermal settings, followed by 17 from alkaline lakes, 13 from mesophilic marine habitats, 10 from mesophilic freshwater habitats, 11 from human-influenced industrial and agricultural environments, and 1 from a sulfidic karst cave (Fig. 1B).

Among the 74 strains with positive experimental evidence of MSD, only 60 have publicly available genome sequences (Table 1, Supplementary table 1). Unfortunately, the genomic sequence from the sole MSD-positive strain of *Pseudomonadota*, *P. agglomerans* SP1 [16], is not available. Further analyses are therefore limited to four phyla of sulfur disproportionators. To the 60 MSD-positive strains with sequenced genomes, we added 12 genomes of strains with no MSD capability after experimental analysis as a negative control, and 39 strains with sequenced genomes, but untested for MSD, from the same four target phyla for taxonomic representativity. This resulted in a database of 111 predicted proteomes for subsequent use in sequence similarity searches (Supplementary table 1).

To simplify the functional diversity of MSD-capable bacteria and to emphasize the importance of S^0^ disproportionation for the search for molecular markers, we divided the strains into five functional categories based on available experimental evidence for or against MSD capabilities: (i) Category "++" includes strains with evidence for S^0^ disproportionation regardless of evidence for or against MSD of sulfite or thiosulfate. (ii) Category " + " includes strains with no evidence for or against S^0^ disproportionation but with positive evidence for MSD of sulfite and/or thiosulfate. (iii) Category "?" includes strains with no evidence for or against MSD whatsoever. (iv) Category "–" includes strains with evidence against MSD of at least 1 sulfur species and no positive evidence. (v) Category "– –" includes strains with evidence against MSD of elemental sulfur, sulfite, and thiosulfate.

### Distribution of microbial sulfur disproportionation candidates

**Figure 2:**
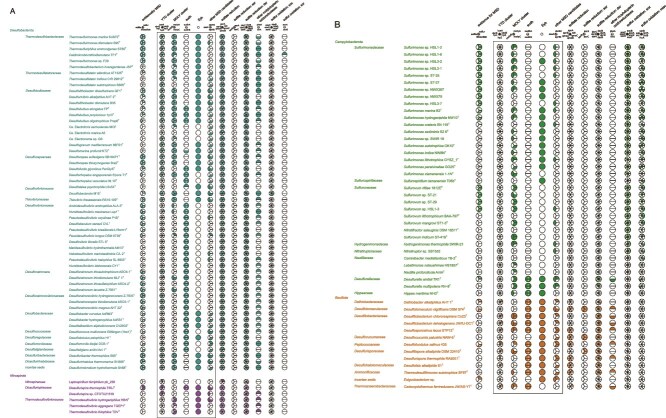
Coulson plot of experimental evidence for MSD and genes and systems searched in the genomes of the strains of interest. In the “evidence for MSD” column, plus indicates positive experimental evidence for disproportionation of the particular sulfur species, minus indicates experimental evidence against it, and empty section indicates lack of any experimental evidence. Colored sections indicate presence of the particular gene. Grey sections indicate presence of a highly divergent homolog. Letters in colored and grey sections indicate syntenic blocks. (A) *Desulfobacterota* and *Nitrospirota*. (B) *Campylobacterota* and *Bacillota*.

HMM profiles were constructed for 57 individual protein-coding genes from these metabolic categories: sulfur disproportionation candidates, genes common to sulfur disproportionation and reduction, genes common to sulfur disproportionation and oxidation, sulfur oxidation, sulfite and sulfur reduction—from alignments of well-characterized publicly available sequences (Supplementary table 2, Supplementary file 1). We searched them using profile hidden Markov models against the 111 proteomes. Results are summarized in Fig. 2, Supplementary file 2, and Supplementary table 1.

One of the candidate proteins investigated in previous work [6] and reported as encoded by all studied *Desulfobacterota* was the short transmembrane protein DsrE2, with a reference to the sequence from *Allochromatium vinosum* (*Gammaproteobacteria*, *Pseudomonadota*) [90]. Our search based on the *A. vinosum* sequence (UniProt D3RPC1) as a seed recovered homologs with the characteristic two transmembrane helices only in *D. amilsii* and *Desulfurella multipotens* (*Desulfurellaceae*, *Campylobacterota*) and in three representatives of *Bacillota*: *Desulfitobacterium chlororespirans* Co23^T^, *D. dehalogenans* JW/IU-DC1^T^, and *Desulfitibacter alkalitolerans* sk.kt5^T^, but only *D. amilsii* has positive experimental evidence for MSD. No member of the DsrE superfamily with two transmembrane helices was found in *Desulfobacterota*. The previously reported genes might represent a different member of the same superfamily [91], possibly the DsrE (UniProt A0A179D5T7) putatively involved in the YTD cluster, which is indeed widespread among *Desulfobacterota*, as discussed below. Therefore, DsrE2 is not a promising candidate for a role in MSD.

The rhodanese-like sulfurtransferases of the SseA superfamily were found distributed across all four investigated phyla in a pattern consistent with that reported in previous work [6] and with no apparent connection to the distribution of positive MSD evidence. Their scattered presence and absence across the MSD-positive strains of the genera *Sulfurimonas* and *Sulfurovum* provides no indication that SseA involvement in MSD could be extended beyond *D. amilsii*, where it was originally suggested [6, 31], even only in *Campylobacterota*.

Previous work [32] proposed a truncated β subunit of APS reductase (AprBt; defined as missing the last 28 residues in the protein sequence of *Desulfovibrio vulgaris*) as a possible molecular marker of MSD. Our search recovered the *aprB* gene in all studied *Desulfobacterota*, some *Nitrospirota* and *Bacillota*, and no *Campylobacterota*, consistently with the rest of the sulfate reduction machinery, as discussed below. Using the same criterion as in the original work [32], we confirm that AprBt is present in all studied representatives of the family *Desulfobulbaceae* (*Desulfobacterota*). Moreover, it is a shared feature of the entire order *Desulfobulbales*, but also the entire order *Desulfobacterales*, and the family *Thermodesulfatatoraceae* within the order *Thermodesulfobacteriales*. Furthermore, the truncated sequence is present in all studied *Bacillota* that have AprB. This pattern is true for all studied representatives of these taxa regardless of their MSD capabilities and thus does not support the proposed use of AprBt as a molecular marker of MSD.

The distribution of the 2-subunit dehydrogenase SudhAB also mostly follows taxonomy rather than MSD capabilities. Within *Desulfobacterota*, both subunits, often in close proximity on the chromosome, were found in almost all studied strains from the orders *Desulfovibrionales* and *Desulfobacterales* and in the genera *Desulfobulbus*, *Desulfofustis*, and *Desulforhopalus* of the order *Desulfobulbales*, whereas they are completely missing from the order *Thermodesulfobacteriales*. Within *Campylobacterota*, both subunits are present in all three studied members of the order *Desulfurellales* and almost entirely missing from the orders *Campylobacterales* and *Nautiliales*. Within *Bacillota*, almost all studied representatives have both subunits. Only in *Nitrospirota* does the distribution suggest a possible connection with MSD, because the unique representative of this group with both subunits, *Dissulfurispira thermophila* T55J^T^, is also the only one known to perform the metabolism [12].

**Figure 3:**
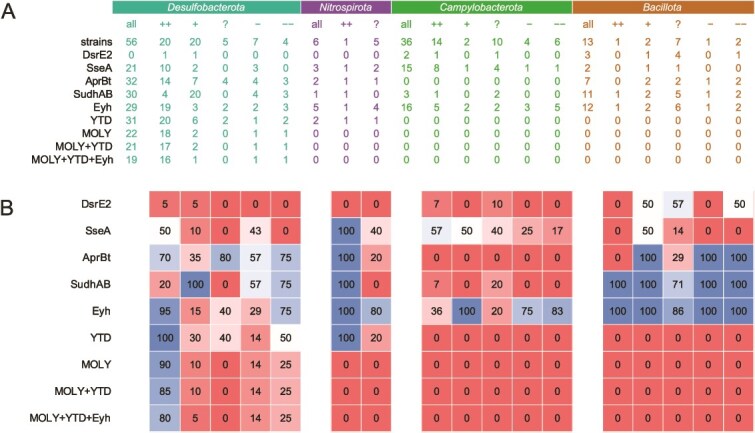
Presence of candidate genes in the strains of interest. (A) Presence of the candidate genes and systems in the functional categories of the strains of interest. (B) Heatmap of relative abundance of the candidate genes and systems in the functional categories of the strains of interest.

The search for Eyh protein sequences often resulted in two paralogs with comparable *E*-values in the same strain. We suspected that one of these might represent FlhB, a bacterial flagellar protein [92] known to be closely related to Eyh [34]. We reconstructed a single-gene phylogenetic tree from all the recovered sequences and indeed, it showed a clear, well-supported split between FlhB and Eyh (Supplementary file 3, Supplementary file 4). Further, we discuss the Eyh sequences only. Again, the distribution of Eyh mostly follows taxonomy and not MSD capabilities. Among *Desulfobacterota*, the gene is present in all members of *Thermodesulfobacteriales* and *Desulfobulbales*, except in the genus *Desulforhopalus* and the "cable bacteria" of the genera *Electrothrix* and *Electronema*, and fully absent from *Desulfovibrionales*. Among *Nitrospirota*, Eyh is present in all five included members of *Thermodesulfovibrionales*. Among *Campylobacterota*, it is present in all three included members of *Desulfurellales* but scattered across the diversity of *Campylobacterales* with no connection to the distribution of MSD, even within the well-characterized genus *Sulfurimonas*. Among *Bacillota*, it is present in all strains except one. This distribution indicates that Eyh cannot be used as a molecular marker of MSD in any of the four phyla. However, its almost ubiquitous presence among *Desulfobacterota* strains able to disproportionate S^0^ (19 of 20, or 95% in category ++) and almost full absence in those disproportionating only sulfite and/or thiosulfate (3 of 20, or 15% in category +) suggests that Eyh might still have a supplementary role specifically in S^0^ MSD in *Desulfobacterota* (Fig. 3).

Searches for genes of the YTD and MOLY clusters often resulted in multiple paralogs with comparable *E*-values. Given that these genes are not well characterized, and their evolutionary relationships are unknown, we were not able to decide on appropriate *E*-value thresholds for inclusion. Especially the individual types of molybdopterin enzymes are difficult to discriminate and no sequence features have been established to identify the enzymes involved in the MOLY cluster. For this reason, we further consider components of the YTD and MOLY clusters as positively identified in the genomes of interest only when belonging to putative orthologous groups (OGs) that contain the sequences using which these clusters were originally defined. This line of evidence is further corroborated by the presence of sequences from full sets of in-synteny genes as discussed below. The OG boundaries presented here are not fully conclusive and certainly can be refined in the future with broader taxon sampling and improved phylogenetic methods.

### Phylogeny of microbial sulfur disproportionation candidates

We reconstructed single-gene trees for each of the genes and annotated them with locations of the paralogs, as well as locations of sequences that form syntenic blocks with other genes of their respective clusters (Supplementary file 3). In the trees of YedE, TusA, MolyA, MolyB, and MolyC, we identified distinct clans that can be identified as putative OGs originating from ancient duplication events. In each of these trees, one OG contained a majority of sequences that come from full sets (YedE, TusA, DsrE, and at least two conserved hypothetical proteins for the YTD cluster and MolyABC for the MOLY cluster) and form at least partial syntenic blocks with their clusters. We marked these clans as OG1 in each tree and further considered only these as positive identifications of the respective genes. For the sake of completeness and reproducibility, genes from other OGs are still included in Supplementary table 1, but they are not considered in further discussions.

The complete YTD cluster is present only in *Desulfobacterota* and *Nitrospirota*, with rather patchy distribution (Fig. 2A). Its presence in all the S^0^ disproportionators, but also some strains with experimental evidence against all forms of MSD (Fig. 3), indicates that it may be involved in S^0^ MSD, but cannot be used as molecular marker on its own. Isolated YedE belonging to OG1 is found also scattered across the diversity of *Bacillota*, but with no apparent connection to the distribution of MSD.

The full MOLY cluster is present only in *Desulfobacterota* with a patchy distribution (Fig. 2). It is found in 90% of the S^0^ disproportionators (category ++) but only in small fractions of other categories (Fig. 3B). Isolated *molyA* gene belonging to OG1 is found also in all members of classes *Thermodesulfovibrionaceae* (*Nitrospirota*) and *Desulfurellaceae* (*Campylobacterota*), likely reflecting the deep-branching positions of these classes within their phyla and the close phylogenetic relationship of *Nitrospirota* and *Campylobacterota* to *Desulfobacterota*.

The chaperone component of the MOLY cluster, MolyC, belongs to the diverse family of Redox Enzyme Maturation Proteins (REMPs) which are all involved in oxidoreductase enzyme maturation by interacting with the Twin-Arginine Translocase (TAT) resulting in secretion of the oxidoreductases across the cytoplasmic membrane [93]. Specifically, MolyC is closely related to the "3f" clade of TorD (Supplementary file 3). This corroborates the extracellular localization of MolyA and MolyB predicted by DeepTMHMM 1.0 (not shown) and their possible involvement in processing extracellular molecules such as elemental sulfur.

MolyAB belongs to the Complex Iron–Sulfur Molybdoenzyme (CISM) family of multi-subunit enzymes [94] which includes other important contributors to sulfur metabolism: thiosulfate reductase PhsAB, best characterized in sulfur-respiring *Gammaproteobacteria* [95] and tetrathionate reductase TtrAB, characterized in *Salmonella typhimurium* (*Gammaproteobacteria*) [96]. In the phylogenetic tree reconstructed for MolyA (Supplementary file 3), the *Gammaproteobacteria* sequences of PhsA branch together in OG3. Therefore, we tentatively annotate all genes belonging to OG3 as PhsA. The well characterized sequences of TtrA branch together in OG9, leading to a tentative annotation of all sequences from this OG as TtrA.

We have found *molyAB* genes scattered across the diversity of studied *Desulfobacterota*, *Nitrospirota*, and *Campylobacterota* (Fig. 2, Supplementary table 1). MolyA can be distinguished from PhsA through phylogeny and also by its association with the REMP MolyC, as discussed above, whereas other CISM family members are typically associated with transmembrane anchor proteins [97].

### Evolutionary history of microbial sulfur disproportionation candidates

The phylogenetic trees of YedE, TusA, and MolyA (Supplementary file 3, Fig. 4A) show multiple putative orthologous groups where the OG1, considered to be the only one representing the MSD-related genes, contains sequences from a broad diversity of bacteria from both *Gracilicutes* and *Terrabacteria*, representing almost the entire extant bacterial diversity. There is no clear evidence that this resulted from LGT, and therefore vertical inheritance from a common ancestor of all, or almost all, bacteria is the most parsimonious explanation for such pattern. That means that these potentially MSD-related genes, components of the YTD and MOLY clusters, originated by gene duplication in a cell that lived before the diversification of *Gracilicutes* and *Terrabacteria* and then spread by vertical inheritance into a broad spectrum of bacterial lineages.

**Figure 4:**
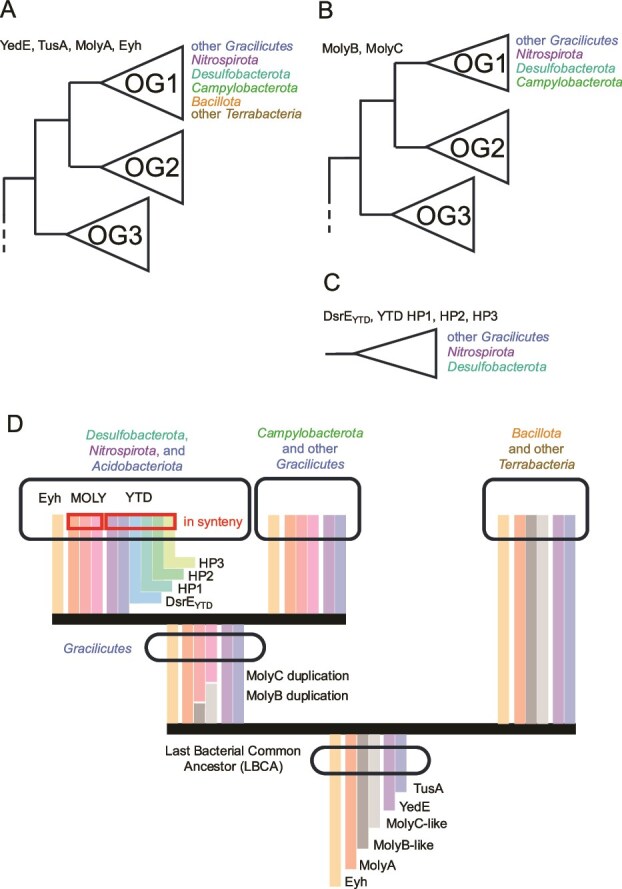
Summary of phylogenetic analyses. (A) Simplified representation of tree topology for genes of interest which have a broad, likely pan-bacterial, distribution of the orthogroup 1 (OG1). (B) Simplified representation of tree topology for genes of interest which have OG1 limited to *Gracilicutes*. (C) Simplified representation of tree topology for genes of interest which have been found only in a subset of phyla from *Gracilicutes*. (D) Schematic representation of the proposed evolutionary scenario where the genes Eyh, MolyA, YedE, and TusA were present already in the last bacterial common ancestor (LBCA), then MolyB and MolyC originated by gene duplication in a common ancestor of *Gracilicutes*, and finally the rest of the YTD cluster components originated by unknown mechanism(s) in a common ancestor of *Desulfobacterota*, *Nitrospirota*, and *Acidobacteriota*.

The trees of MolyB and MolyC (Supplementary file 3, Fig. 4B) show a similar pattern, but OG1 contains almost exclusively members of *Gracilicutes*, the group that includes *Desulfobacterota*, *Nitrospirota*, *Campylobacterota*, but also *Pseudomonadota*, *Spirochaetota*, *Bacteroidota*, and many other lineages. It does not include *Bacillota* which have MSD-positive representatives. Again, there is no evidence for LGT in the trees. This pattern is most parsimoniously explained by the origin of the potentially MSD-related *molyB* and *molyC* genes by gene duplication in a common ancestor of *Gracilicutes* which lived after the separation of *Gracilicutes* and *Terrabacteria*.

Four remaining proteins of the YTD cluster, DsrE and three hypothetical conserved proteins CHP1–3, have been found only in three phyla, namely *Desulfobacterota*, *Nitrospirota*, and *Acidobacteriota*. There is no evidence for LGT in the phylogenetic trees (Supplementary file 3, Fig. 4D) and these three phyla are closely related to each other, which supports the parsimonious scenario of all four genes having their origin (by gene duplication or otherwise) in a common ancestor of *Desulfobacterota*, *Nitrospirota*, and *Acidobacteriota*.

These phylogenetic patterns of MOLY and YTD clusters (summarized schematically in Figs 4A–C) are consistent with an evolutionary scenario (Fig. 4D) where both clusters originated in their present configurations in a common ancestor of *Desulfobacterota*, *Nitrospirota*, and *Acidobacteriota*. However, alternative interpretations are possible, because the single-gene phylogenetic trees presented here are necessarily subject to long branch attraction and other phylogenetic artifacts.

### Other pathways of dissimilatory sulfur metabolism

As expected [25, 98], the core components of the dissimilatory sulfite and sulfate reduction machinery (DsrABCKM, AprAB, QmoAB) were found consistently across the diversity of studied *Desulfobacterota*, in *Thermodesulfovibrionales* within *Nitrospirota*, and more disparately across *Bacillota*. The only representatives of *Campylobacterota* to encode components of these pathways were *Desulfurellales*, which are known to reduce sulfite and which were previously classified as *Deltaproteobacteria*, together with the typically sulfate-reducing *Desulfobacterota* [99].

As previously observed [100, 101], genes of the sulfur-oxidizing multienzyme complex Sox, governed by the conserved operon *soxXYZABCG*, were found in almost all *Campylobacterales* but not in any *Desulfurellales* or *Nautiliales* within *Campylobacterota*. This latter result is congruent with physiological observations showing that *Desulfurellales* and *Nautiliales* generally use sulfur compounds as electron acceptors in reduction reactions or as substrates for disproportionation [13, 102]. SoxABXYZ is also encoded in the genome of the recently isolated *Desulfobacterota* strain M19^T^, which represents a new genus and species, the only example among *Desulfobacterota* in our dataset [89]. These results show that the *Desulfobacterota* and *Nitrospirota* studied, which are all (with a single exception of *Leptospirillum ferriphilum*) capable of reducing sulfur compounds, encode the sulfate-reduction pathway (Fig. 2A). This pathway is involved in the oxidative branch of sulfur compound disproportionation in some taxa of these lineages [44]. A complete sulfate reduction pathway is encoded in the genome of certain sulfur-disproportionating taxa unable to reduce sulfate [103–106].

The *Campylobacterota* studied here, described as capable of oxidizing sulfur compounds and sometimes reducing elemental sulfur, do not encode the sulfate reduction pathway, indicating that these strains use other enzymes for their oxidative branch of MSD. Exceptions to this can be found in *Desulfurellaceae* (*Campylobacterota*) which possess a minimal DsrABCNK set similar to the presumably ancestral archaeal pathway of sulfite reduction which later gave rise to the more complex pathway of sulfate reduction by incorporating Sat and AprAB [25]. A comparative proteomic study of *D. amilsii* found no connection between its Dsr proteins and MSD [31]. These observations support the existence of more than one pathway for MSD. It can be hypothesized that MSD pathways are resisting description because they employ enzymes from other catabolic pathways of the sulfur cycle, including molybdopterin oxidoreductases of the CISM family, which cannot be discriminated based on sequence information alone, and which are used either independently, or in combination with enzymes specific to MSD.

### Evolutionary relationships between microbial sulfur disproportionation and microbial sulfate reduction

Sulfur metabolisms were likely among the first to appear on Earth, during the Early Archean era [21, 107, 108]. The history of MSD is clearly linked to that of microbial sulfate reduction (MSR) in lineages such as *Desulfobacterota* and *Nitrospirota*, which raises the question of whether this metabolism appeared before or after MSR on Earth. The Archean Earth displayed a reductive environment and a small reservoir of volcanic sulfur (in the micromolar range) comprising sulfur compounds involved in MSR (sulfate) and MSD (thiosulfate, sulfite, and S^0^) was present [108, 109], meaning that either or both of these metabolisms could theoretically have arisen during this era in specific niches.

Numerous studies have found isotopic evidence of MSR in a variety of Archean rocks [107], and a few studies have also interpreted sulfur isotope ratios in rocks and organic matter from the early Archean as being related to MSD [21, 22, 110], but the antiquity of MSD remains difficult to pinpoint, especially because very few data are available about isotopic fractionation effects associated with MSD [111]. Given the uncertainties and limitations in interpreting geological record, continuing to study the full range of MSD isotope effects, while conducting parallel phylogenetic and molecular clock analyses of sulfur cycling gene events, will be critical for reconstructing the evolutionary history of MSD in relation to MSR.

Large-scale phylogenetic analyses of sulfur cycle enzymes suggest an early origin of sulfite reduction in Archaea followed by a lateral gene transfer to Bacteria, where the sulfate pathway originated and evolved mostly vertically [25, 112]. At this point, it is not possible to discriminate Dsr proteins involved in MSR from those involved in MSD based on genomics alone [25], which does not allow to rule out the appearance of MSD in the early Archean. In addition, as discussed here, numerous strains able to perform MSD encode a full MSR enzymatic machinery but are unable to respire sulfate [103–106].

We demonstrate here that the most promising MSD molecular markers, the YTD and MOLY clusters in *Desulfobacterota* and *Nitrospirota* likely already existed in the last common ancestor of these phyla 3.5 billion years ago, which, if these clusters are specific to MSD, could be a further indication of the very ancient emergence of MSD. However, the birth of a gene does not necessarily coincide with the moment when the function of that gene became ecologically relevant.

The ability to disproportionate elemental sulfur has also been demonstrated in the phylum *Bacillota*, which is among the oldest bacterial phyla [35, 113]. However, the presence of a physiological trait in an ancient lineage does not necessarily indicate that this trait has coevolved throughout geological history. Similarly, the widespread presence of a metabolism in many distant genomes is not in itself proof of its ancestral origin [114].

Based on current knowledge, it is still uncertain whether MSD preceded or followed the emergence of sulfite/sulfate reduction on Earth. Given that MSD uses enzymes from other catabolic pathways of the sulfur cycle, including sulfate reduction enzymes [19], the two possible scenarios would be (i) that MSD arose at the same time as MSR and other sulfur cycle pathways, followed by numerous instances of pathway loss, or (ii) that MSD arose repeatedly during the coevolution of the early Earth and sulfur metabolisms, following multiple events of convergent evolution and the reuse of known proteins in new combinations to perform a different function (MSD), as previously proposed for the order *Desulfobulbales* [57].

To determine when MSD became ecologically relevant on Earth, we must pursue our efforts to characterize the multiple pathways of MSD, consider their coupling to the iron and nitrogen cycles, determine the extent of MSD in the microbial tree of life, determine the full range of MSD isotope effects, and continue studying evolutionary relationships and differences between microbial MSD and other sulfur metabolisms.

## Conclusion

We present a comprehensive survey of all known bacterial strains with experimental evidence for MSD, summarizing their metabolic capabilities, taxonomic position, environment, and genome availability. To this date, MSD was demonstrated in five phyla: *Desulfobacterota* (50 strains), *Nitrospirota* (1 strain), *Campylobacterota* (16 strains), *Bacillota* (6 strains), and *Pseudomonadota* (1 strain). Out of these, 60 strains have their genomes sequenced and published. The MSD-positive strains were isolated from various low-oxygen terrestrial habitats from all continents except Australia and Antarctica and from low-oxygen marine habitats in the Atlantic, Indian, and Pacific oceans, the most common being hydrothermal systems, both deep-sea and shallow. This may be related to the abundant presence of sulfide-scavenging minerals at hydrothermal sites, which increases the energy yield of MSD [10].

Our search for previously proposed molecular markers of MSD revealed that none of the genes, nor any particular combination, can be used as unambiguous molecular marker of these metabolic capabilities. Neither the short transmembrane protein DsrE2, the rhodanese-like sulfurtransferases of the SseA superfamily, nor the truncated version of the adenylylsulfate reductase AprB appear to have distribution patterns compatible with a widespread role in MSD. Among *Desulfobacterota*, the distribution patterns of the 2-subunit dehydrogenase SudhAB and the short uncharacterized protein Eyh suggest possible roles in sulfite and/or thiosulfate disproportionation and elemental sulfur disproportionation, respectively. The best candidates for molecular mechanisms of MSD appear to be the two multi-gene clusters YTD and MOLY. This is partially corroborated by recent findings of the overexpression of the full or partial YTD cluster during MSD in three strains of *Desulfobacterota* ([28, 29] and *M. Fernandez*, personal communication).

No specific combination of genes can be connected to MSD ability across all four investigated phyla. This is consistent with the current understanding that multiple independent mechanisms of independent origins are the best explanation for the extant diversity of MSD-positive bacteria. Based on this work, we can start speculating about the number and distribution of such mechanisms. *Desulfobacterota* and the closely related *Desulfurispora thermophila* (*Nitrospirota*) might utilize a common mechanism involving enzymes otherwise responsible for sulfate reduction, plus the YTD cluster. *D. amilsii* (*Campylobacterota*) might utilize a mechanism based on its Dsr-based sulfite-reducing pathway, with possible involvement of rhodanese-like sulfurtransferases, completely distinct from a mechanism in other MSD-capable *Campylobacterota* within the genera *Sulfurimonas* and *Sulfurovum*, likely utilizing their SorAB-based sulfur oxidation pathways. Mechanisms responsible for MSD in the four *Bacillota* strains remain obscure.

Based on the distribution and phylogeny of the genes constituting the YTD and MOLY clusters, it is most parsimonious that these clusters, in their current form, existed already in the last common ancestor of *Desulfobacterota*, *Nitrospirota*, and *Acidobacteriota*, as early as 3.5 billion years ago, according to latest molecular dating estimates [112, 113]. This timing is consistent with the earliest point of MSD origin suggested by paleontological evidence [21, 22].

Based on our annotations of other metabolic pathways in the strains of interest, we conclude that MSD-positive bacteria most often possess other sulfur-related catabolic pathways consistently with their taxonomic context such as sulfate reduction in *Desulfobacterota* and *Nitrospirota*, sulfite reduction in *Desulfurellaceae* (*Campylobacterota*), and sulfur oxidation in *Campylobacterales* (*Campylobacterota*). These observations suggest that MSD could be a way for microorganisms involved in the sulfur cycle to escape the physiological and energetic limitations of a single redox couple and colonize a wider range of ecological niches, using, at least in some cases, part of the existing genomic setup of sulfur metabolism.

Direct genetic, biochemical, and enzymatic characterization is needed to support or reject the role of various candidate genes in MSD. Based on the indirect evidence available so far and gathered in this work, we propose that the best candidates for such experiments are the YTD and MOLY clusters, and possibly also Eyh and SudhAB proteins. Further genomic sequencing and physiological experiments can be used to fill gaps in the presented data.

## Supplementary Material

Sup_File_1_HMM_wrag042

Sup_File_2_synteny_wrag042

Sup_File_4_raw_trees_wrag042

Sup_File3_trees_wrag042

Sup_Tab_1_strains_wrag042

Sup_Tab_2_sequences_wrag042

Sup_Tab_3_constraints_wrag042

Supplementary_captions_wrag042

## Data Availability

The genomes analyzed in this study are available in the NCBI GenBank and IMG/M repositories and are listed in Table 1.
