# Supplementary material for: Sulfur disproportionation occurs globally across anoxic habitats and has multiple mechanisms of independent evolutionary origin
Source: ISME J. 2026 Mar 2;20(1):wrag042. doi: 10.1093/ismejo/wrag042 (PMC12998232; doi:10.1093/ismejo/wrag042)
Supplement: Supplementary_captions_wrag042 [file supplementary_captions_wrag042.docx]

**Supplementary table 1**: Strains of interest with experimental evidence for MSD and IDs of genes identified in this work.

**Supplementary table 2**: Seed sequences used in phylogenetic analyses.

**Supplementary table 3**: Taxonomic constraints for BLASTp searches used in constructing phylogenetic datasets.

**Supplementary file 1**: HMM profiles used in the study, including the full sequence datasets of newly constructed profiles, and output of all HMM searches.

**Supplementary file 2:** Arrangement of the discussed syntenic blocks in the genomes of the strains of interest. Arrows indicate the orientation of the individual genes within the published genomic assemblies. Black lines connecting the arrows indicate immediate physical relationship between the genes on a contig. Breaks in the black line indicate either breaks in the contigs or a substantial distance within the same contig (separation by more than 10 open reading frames). MSD candidate genes are assigned individual colors; orange arrows indicate genes involved in reduction of sulfur species; green arrows indicate genes involved in oxidation of sulfur species; grey arrows indicate interleaving genes that are not discussed in this work.

**Supplementary file 3**: Single-gene phylogenetic trees of the genes of interest. Grey and white background: putative OGs. Cyan background: *Desulfobacterota*, magenta background: *Nitrospirota*, green background: *Campylobacterota*, orange background: *Bacillota*, blue text: other *Gracilicutes*, brown text: other *Terrabacteria,* black text: other *Bacteria* and *Archaea*. Red text: paralogs used for putative OGs delimitation.

**Supplementary file 4**: Phylogenetic alignments and Newick format single-gene trees.
