# Supplementary material for: Sulfur disproportionation occurs globally across anoxic habitats and has multiple mechanisms of independent evolutionary origin
Source: ISME J. 2026 Mar 2;20(1):wrag042. doi: 10.1093/ismejo/wrag042 (PMC12998232; doi:10.1093/ismejo/wrag042)

Eyh

ML, IQtree, LG+C60+F+G, ultrafast BS / SH-like approximate likelihood ratio test*

* only ≥50% shown, only on the backbone and Eyh

1 2 putative orthologous groups

Desulfobacterota | *Bacillota*

Nitrospirota | other *Terrabacteria*

Campylobacterota | other *Bacteria* and *Archaea*

other *Gracilicutes* | ← **paralogs**

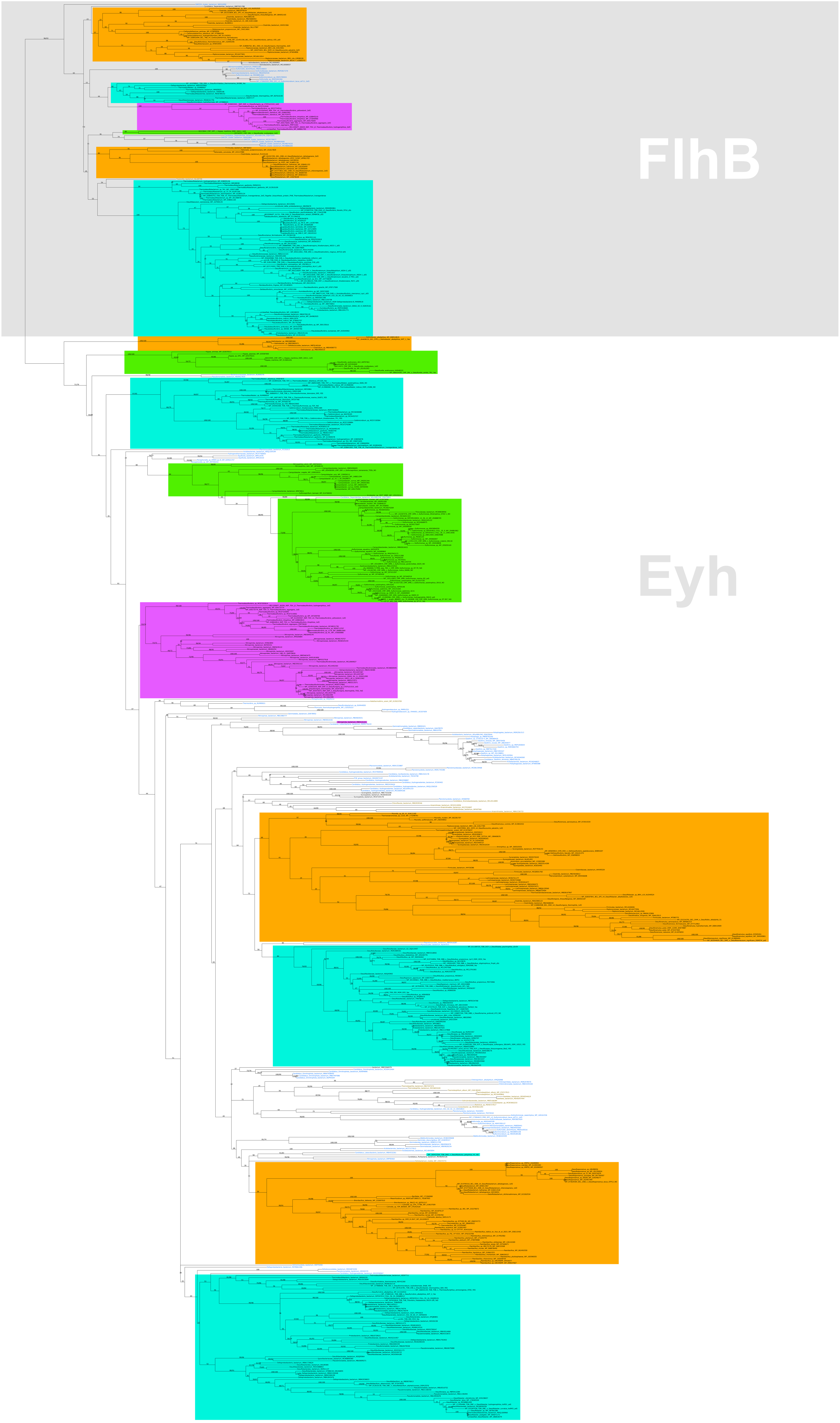

YedE (YTD cluster)

ML, IQtree, LG+C60+F+G, ultrafast BS / SH-like approximate likelihood ratio test*

* only ≥50% shown

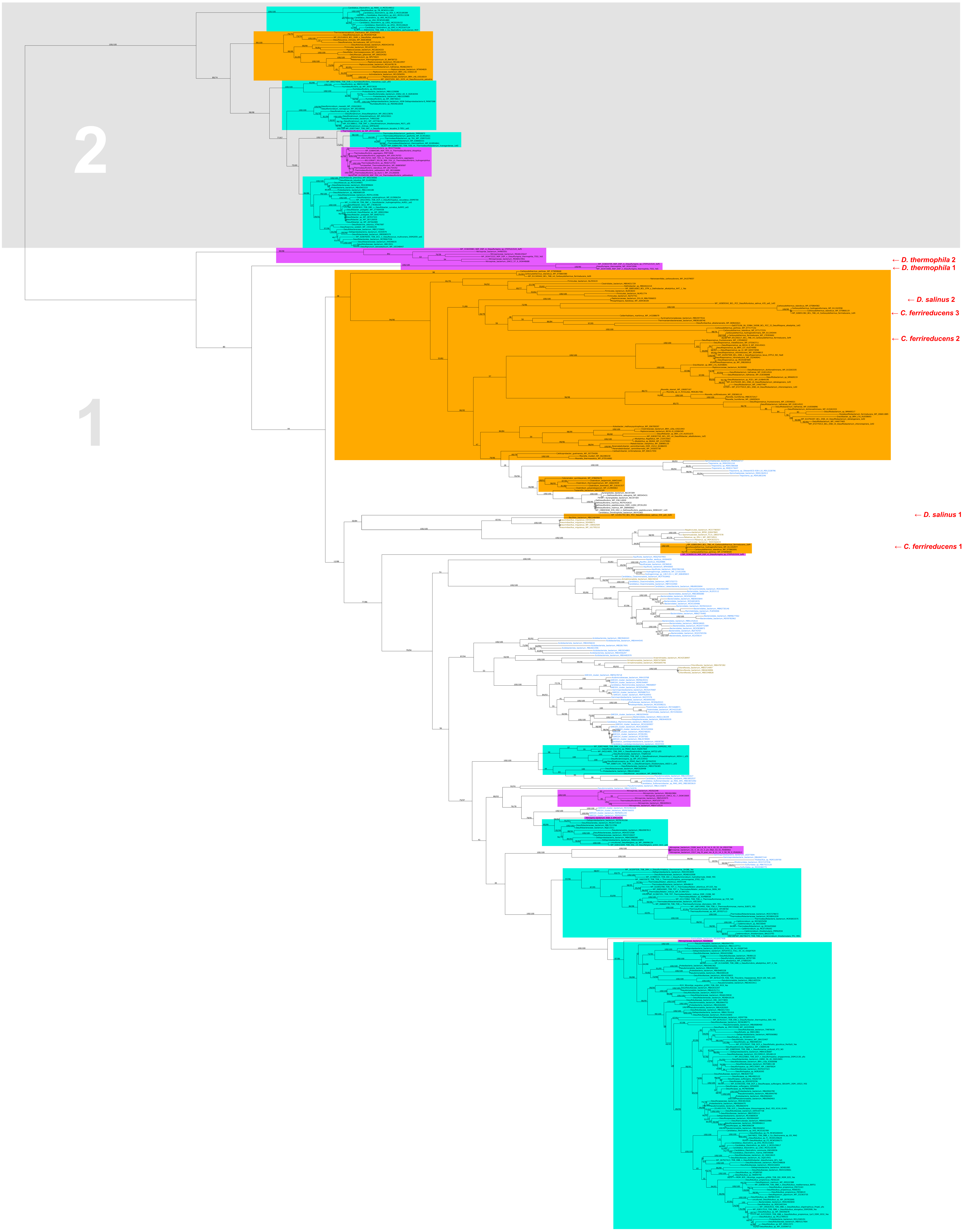

TusA (YTD cluster)

ML, IQtree, LG+C60+F+G, ultrafast BS / SH-like approximate likelihood ratio test*

* only ≥50% shown

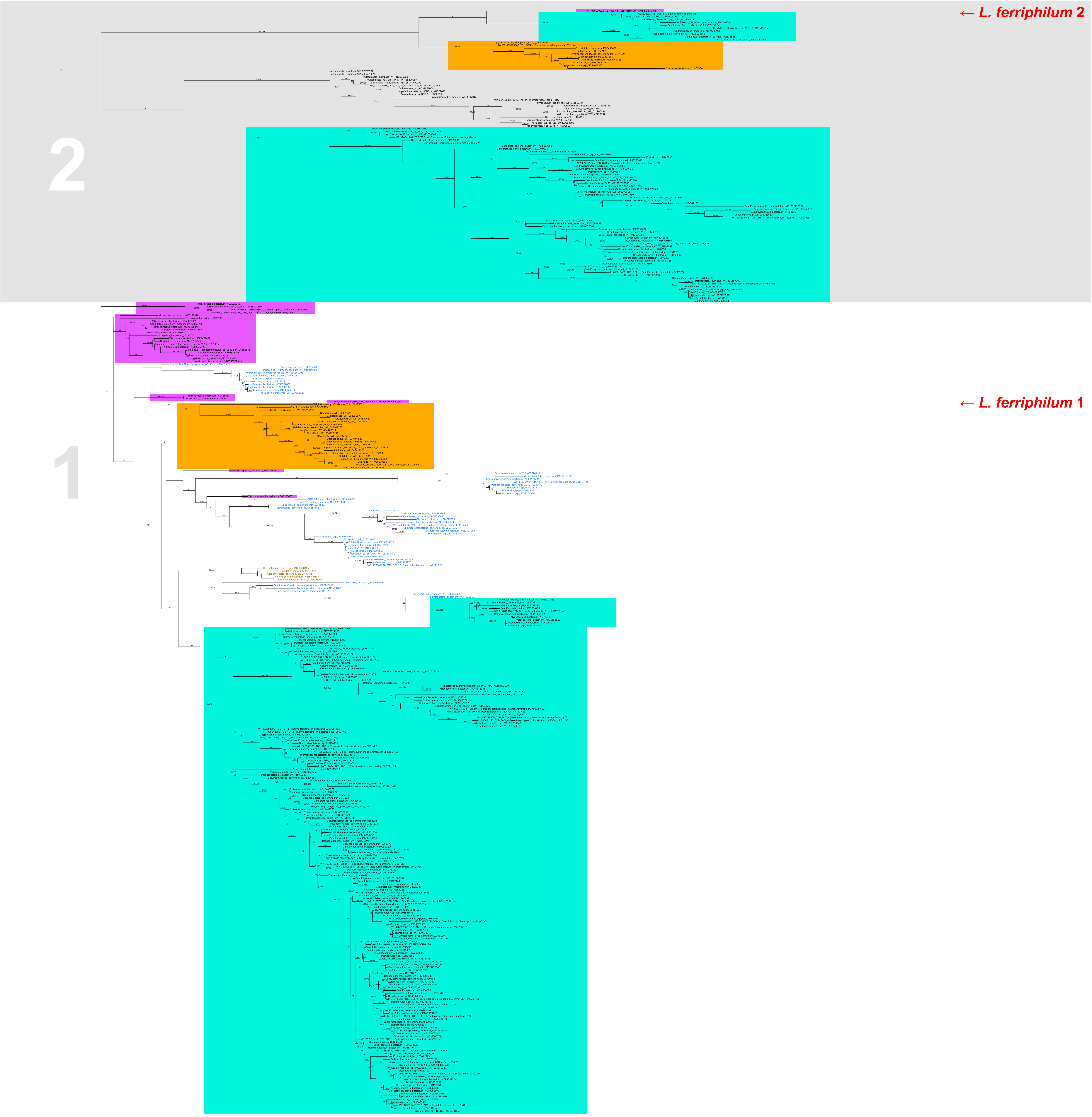

DsrE (YTD cluster)

ML, IQtree, LG+C60+F+G, ultrafast BS / SH-like approximate likelihood ratio test*

* only ≥50% shown

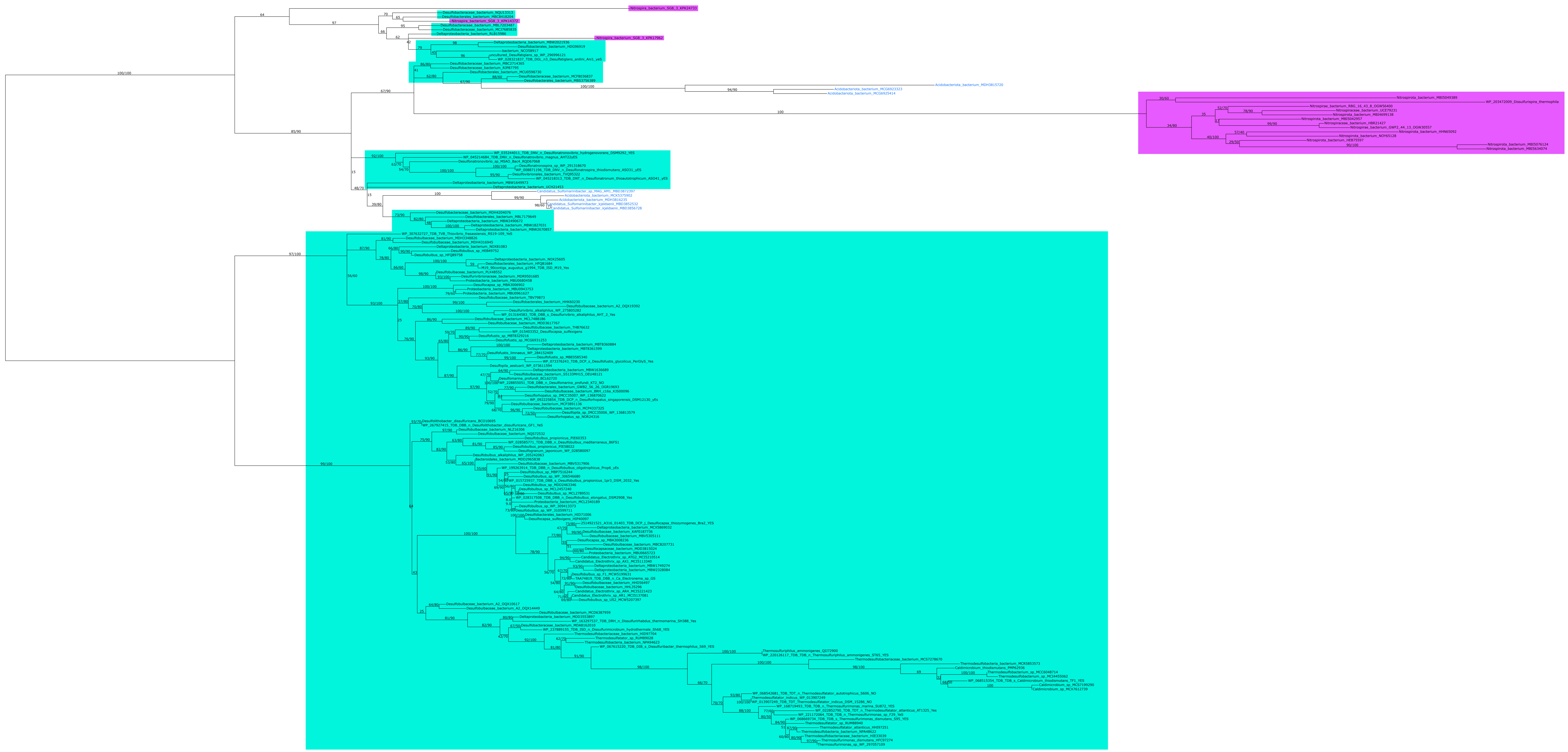

CHP1 (YTD cluster)

ML, IQtree, LG+C60+F+G, ultrafast BS / SH-like approximate likelihood ratio test*

* only ≥50% shown

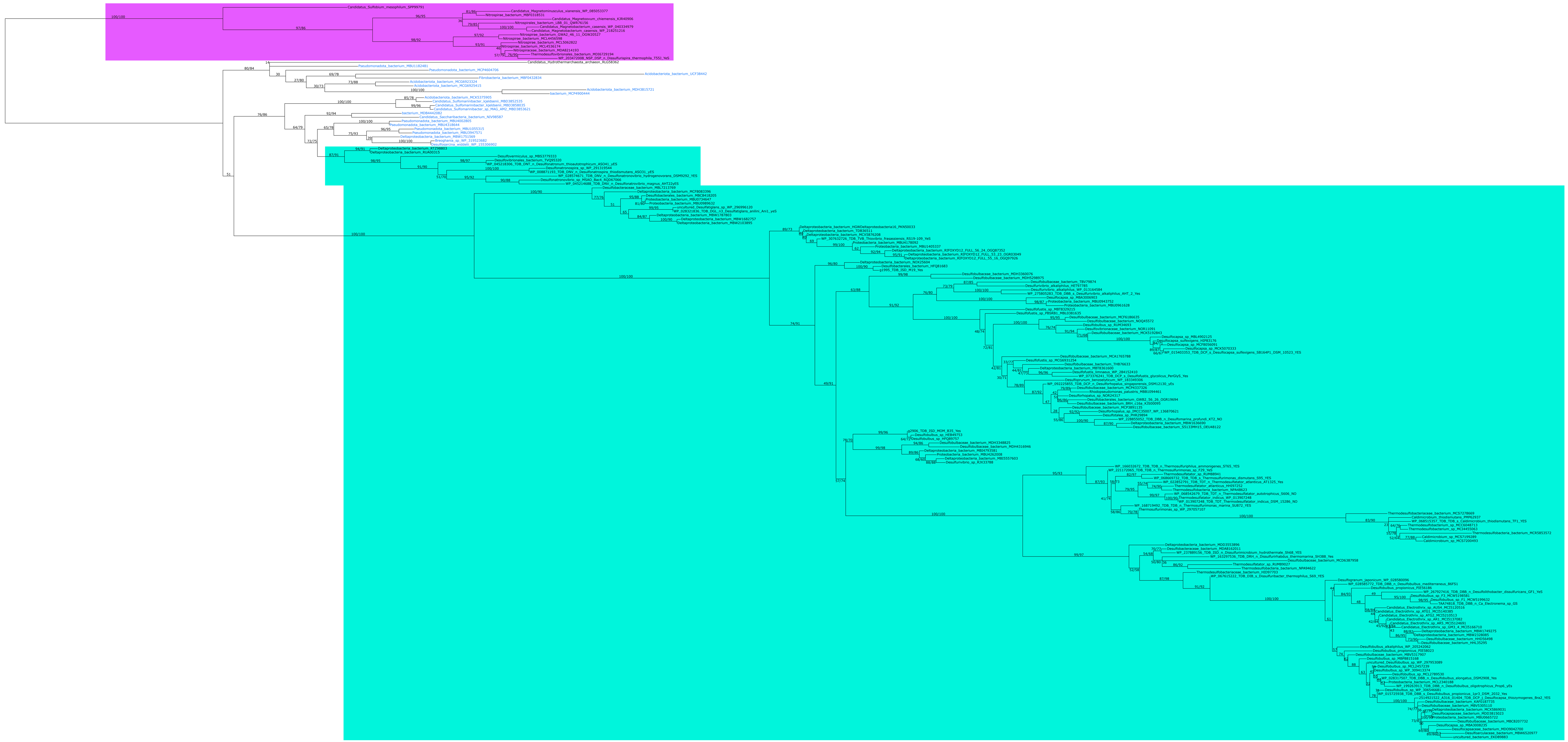

CHP2 (YTD cluster)

ML, IQtree, LG+C60+F+G, ultrafast BS / SH-like approximate likelihood ratio test*

* only ≥50% shown

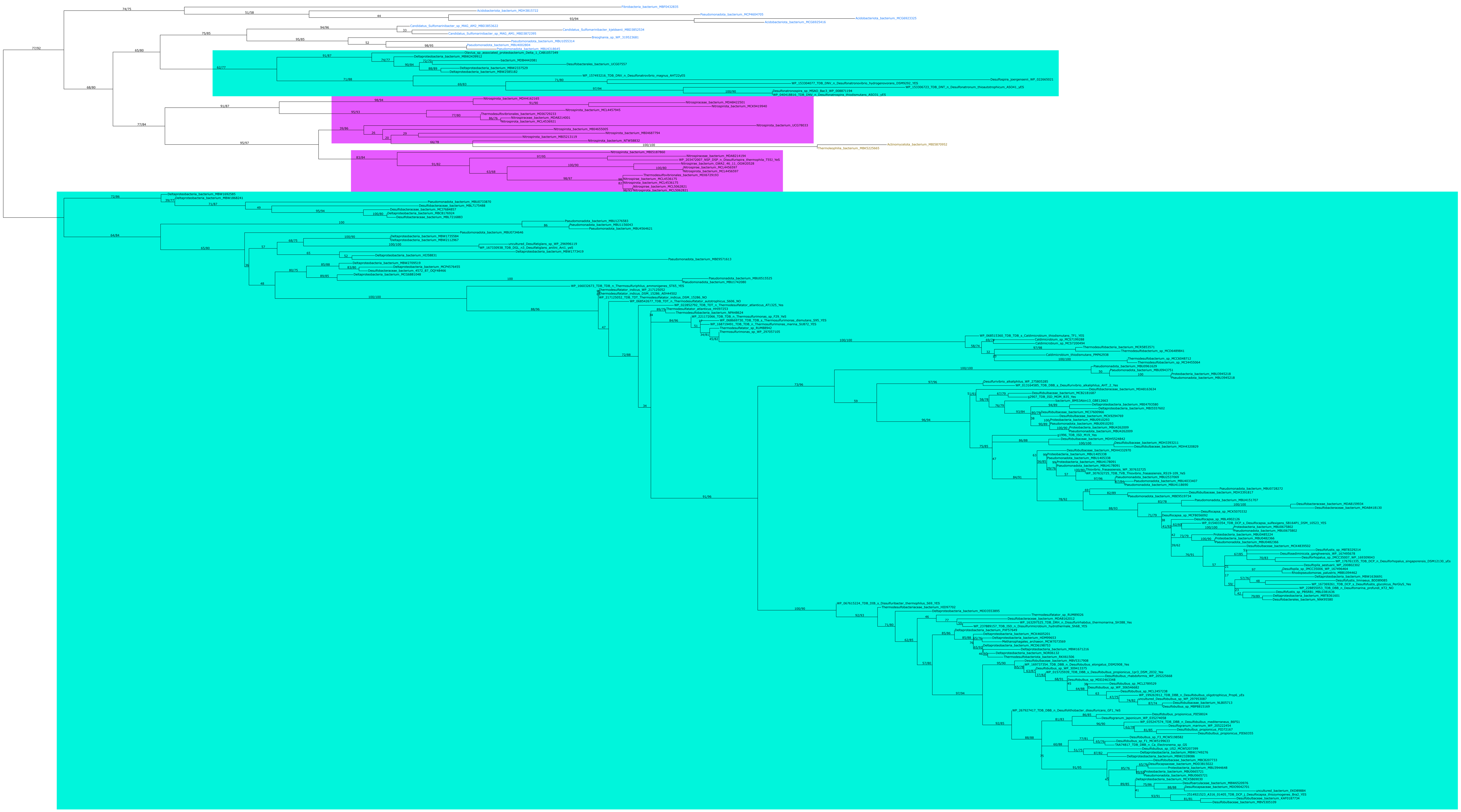

CHP3 (YTD cluster)

ML, IQtree, LG+C60+F+G, ultrafast BS / SH-like approximate likelihood ratio test*

* only ≥50% shown

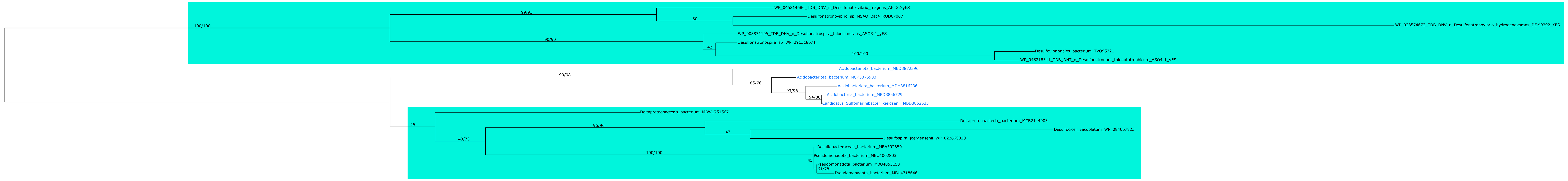

MolyA (MOLY cluster)

ML, IQtree, LG+C60+F+G, ultrafast BS / SH-like approximate likelihood ratio test*

* only ≥50% shown, only on the backbone and OC1

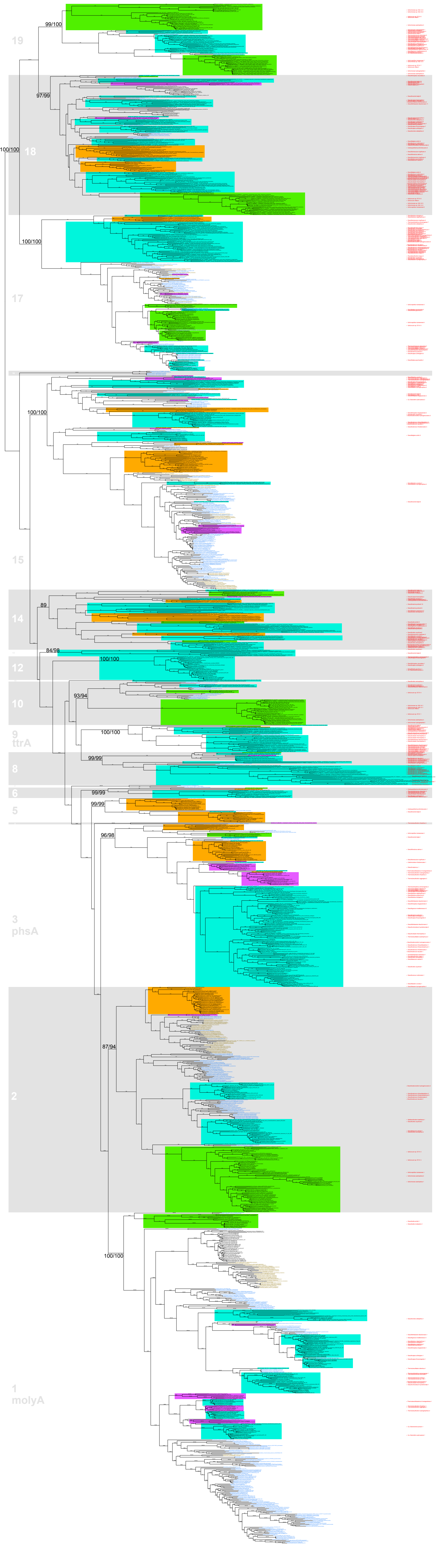

MolyB (MOLY cluster)

ML, IQtree, LG+C60+F+G, ultrafast BS / SH-like approximate likelihood ratio test*

* only ≥50% shown, only on the backbone and OC1

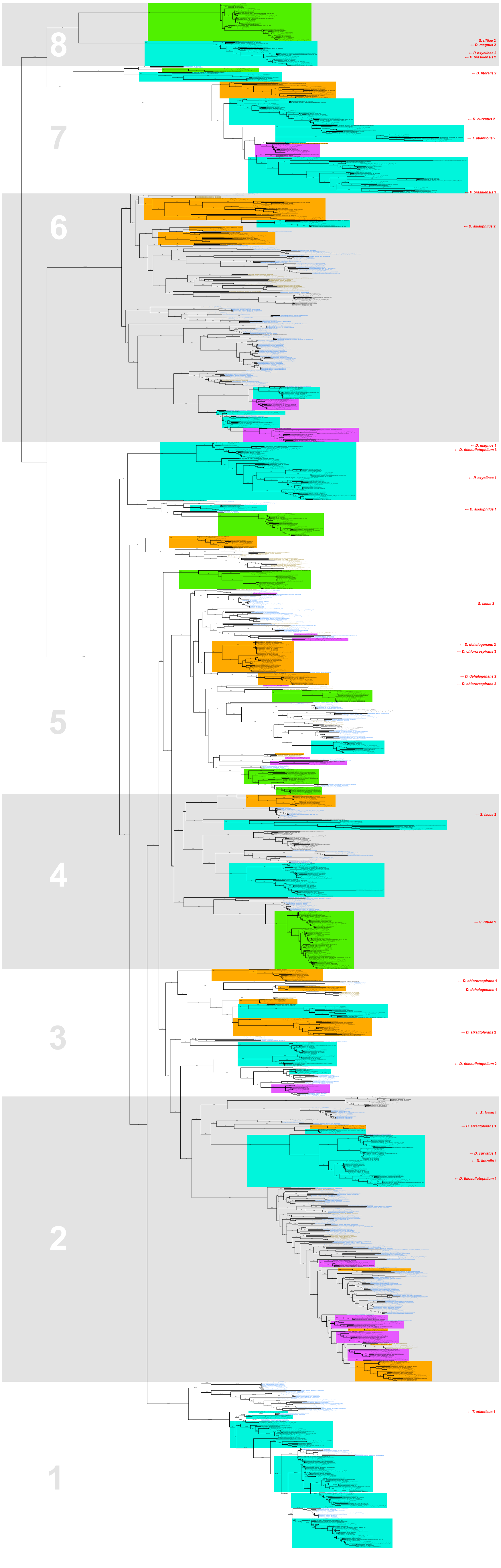

MolyC (MOLY cluster)

ML, IQtree, LG+C60+F+G, ultrafast BS / SH-like approximate likelihood ratio test*

* only ≥50% shown, only on the backbone and OC1

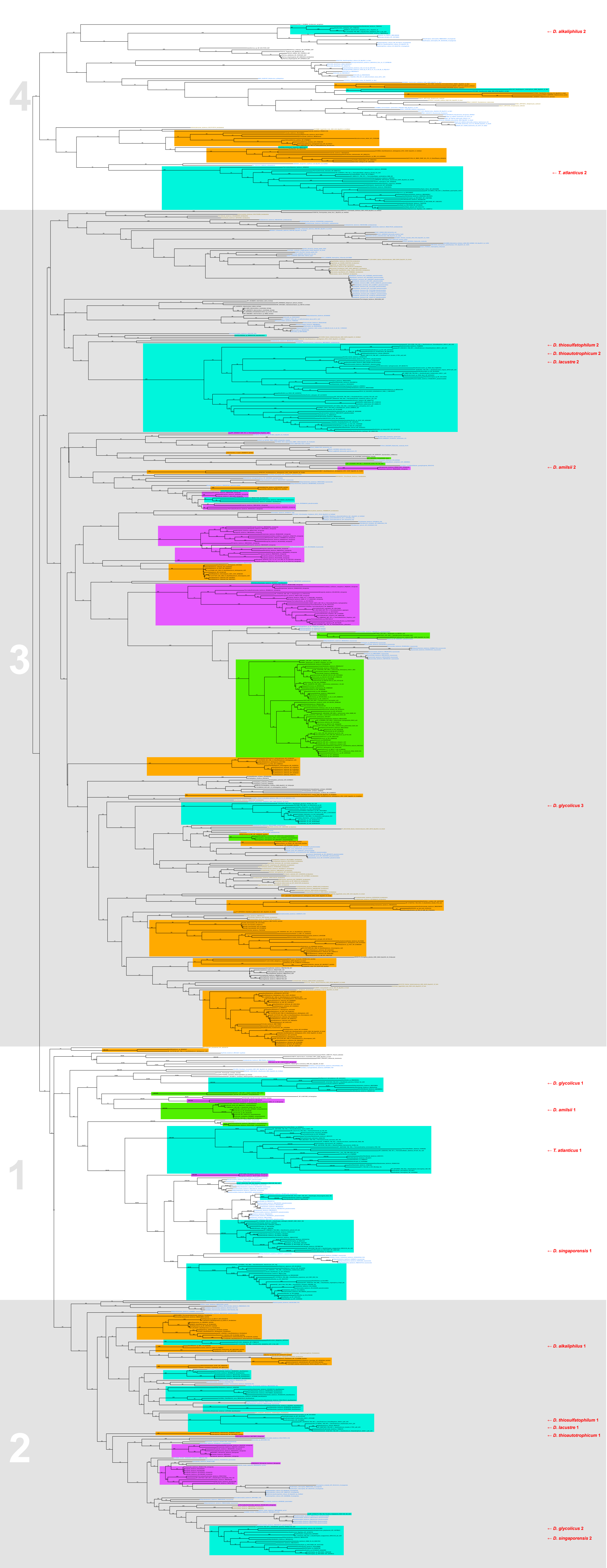

Supplement: Sup_File3_trees_wrag042 [file sup_file3_trees_wrag042.pdf]
